# Management practices in facilities providing HIV services to key populations in Kenya and Malawi: A descriptive analysis of management in community-based organizations

**DOI:** 10.1371/journal.pgph.0002813

**Published:** 2024-03-20

**Authors:** Andrea Salas-Ortiz, Marjorie Opuni, Alejandra Rodríguez-Atristain, José Luis Figeroa, Jorge Eduardo Sánchez-Morales, Agatha Kapatuka Bula, Barbara Nyambura, Sergio Bautista-Arredondo

**Affiliations:** 1 Centre for Health Economics, The University of York, York, England; 2 Independent Public Health Researcher, Lausanne, Switzerland; 3 Division of Health Economics and Health Systems Innovations, National Institute of Public Health (INSP), Cuernavaca, Mexico; 4 UNC Project-Malawi, Lilongwe, Malawi; 5 UNC School of Nursing, The University of North Carolina at Chapel Hill, Chapel Hill, North Carolina, United States of America; 6 Independent Public Health Consultant, Nairobi, Kenya; University of Alberta, CANADA

## Abstract

HIV services for key populations (KP) at higher risk of HIV infection are often delivered by community-based organizations. To achieve HIV epidemic control, countries need to scale up HIV services for KP. Little is known about the management practices of community-based organizations delivering health services. We explored the management practices and facility characteristics of community-based health facilities providing HIV services to key populations as part of the LINKAGES program in Kenya and Malawi. We collected information on management practices from 45 facilities called drop-in centers (DICs) during US Government FY 2019, adapting the World Management Survey to the HIV community-based health service delivery context. We constructed management domain scores for each facility. We then analyzed the statistical correlations between management domains (performance monitoring, people management, financial management, and community engagement) and facility characteristics (e.g., number of staff, organization maturity, service scale) using ordinary least square models. The lowest mean management domain scores were found for people management in Kenya (38.3) and financial management in Malawi (25.7). The highest mean scores in both countries were for performance monitoring (80.9 in Kenya and 82.2 in Malawi). Within each management domain, there was significant variation across DICs, with the widest ranges in scores (0 to 100) observed for financial management and community involvement. The DIC characteristics we considered explained only a small proportion of the variation in management domain scores across DICs. Community-based health facilities providing HIV services to KP can achieve high levels of management in a context where they receive adequate levels of above-facility support and oversight—even if they deliver complex services, rely heavily on temporary workers and community volunteers, and face significant financial constraints. The variation in scores suggests that some facilities may require more above-facility support and oversight than others.

## Introduction

Together with their partners, key populations at higher risk of HIV infection—including men who have sex with men, female sex workers, people who inject drugs, and transgender women—are estimated to account for 70% of new HIV infections globally [1]. In sub-Saharan Africa, new infections among key populations and their partners make up 74% of new HIV infections in West and Central Africa and 46% in Southern and Eastern Africa and there is growing concern that as HIV epidemics in the general population are better controlled, the relative importance of key populations will increase [1–3]. To achieve HIV epidemic control, countries around the world, including in sub-Saharan Africa, will need to scale up HIV services for key populations [2, 3]. Effective HIV services for key populations are often delivered using community-based approaches that increase the accessibility and acceptability of services [4, 5]. Evidence is urgently needed on the organizational characteristics of the community-based organizations delivering HIV services to key populations, the constraints they face, and the best solutions to these limitations.

There is growing evidence that there are good and bad management practices [6, 7] and that effective management is an essential feature of health facility performance [8, 9]. Studies in high-income countries have shown that hospitals with higher management scores are more productive [10, 11] and have better quality of care [11–15]. A few studies in primary healthcare facilities in low-resource countries have also shown that facilities with higher management scores outperformed those with lower management scores [16–18]. One study in health facilities providing HIV services in low-resource countries found that some management practices were associated with better facility performance [19], while another study found no statistically significant relationship [20].

However, none of the facilities included in these studies were owned or operated by community-based organizations, and little is known about the management practices and the management capacity of these organizations. Two qualitative studies in the United States found low levels of management capacity in community-based organizations providing HIV services [21, 22]. A recent qualitative study of management practices among community-based organizations providing HIV services to female sex workers in Nigeria also found that management capacity was limited, though long-standing organizations had better management capacity compared with fledgling ones, especially in HIV service delivery planning [23].

Community-based health facilities providing HIV services to key populations may pose management challenges compared to government or for-profit health facilities that provide HIV services to the general population. The services delivered are more complex, comprising both clinical services and empowerment and engagement activities, structural interventions (e.g., stigma reduction activities), and peer outreach [24–29] and services are delivered at fixed sites as well as directly in communities [27–29]. At the same time, the cadre of service providers varies across organizations and includes many temporary workers and community volunteers. Staff and volunteers often need substantial support, including program management support, technical assistance, training, and oversight from individuals and institutions above the service level. Moreover, community-based organizations usually face unique funding constraints [30] and funding often comes from multiple sources, with varying performance monitoring and reporting requirements [31].

In this paper, we explore the management practices of community-based health facilities delivering HIV services to key populations as part of the Linkages Across the Continuum of HIV Services for Key Populations Affected by HIV (LINKAGES) program in Kenya and Malawi. LINKAGES was a program funded by the United States Agency for International Development (USAID) through the United States President’s Emergency Plan for AIDS Relief (PEPFAR) from 2014 to 2021 and administered by FHI 360. The goals of the program—implemented in countries in Africa, Asia, and the Caribbean—were to reduce HIV transmission among key populations and improve their enrollment and retention in care and treatment services. HIV services were delivered by local community-based organizations called implementing partners (IPs). IPs provided services in facilities called drop-in centers (DICs) and in communities. This paper contributes to the growing literature on management practices in facilities delivering health services in resource-limited settings. We provide information on the management practices of a particular kind of organization—community-based organizations providing HIV services to key populations. We also seek to identify facility characteristics that could be associated with differences in management practices across these organizations.

## Materials and methods

### Management framework

Questions on facility-level management practices were developed based on previous work measuring management in health facilities. We started with the World Management Survey (WMS) framework developed to measure management in manufacturing firms [7] and applied to healthcare facilities in high-income countries [11–14]. The WMS focuses on management practices in four domains: target setting, performance monitoring, people management, and operations management [32–34]. Target setting refers to setting appropriate targets, tracking correct outcomes, and ensuring that targets and outcomes align. Performance monitoring describes the collection and analysis of data to understand an organization’s performance and identify opportunities for improvement. People management includes the various activities related to hiring, retaining, and rewarding high performance and addressing underperformance. Operations management refers to the extent to which processes in the organization are standardized and operations are continuously improved. We also added two additional domains essential to quality management of health facilities in low-resource settings [35]: financial management [8] and community engagement [8, 18]. Financial management describes the budgeting and financial accounting of revenues and expenses to ensure the smooth operations of an organization [8, 35]. Community engagement includes the development and management of relationships with community members through community outreach, involvement of community leaders, and activities to ensure customer trust and satisfaction [8, 35]. We developed questions on management practices in these six domains relevant to community-based health facilities delivering HIV services to key populations in low-resource settings. Unlike the WMS, which uses an in-depth qualitative methodology that is labor intensive and expensive to execute [36], we developed closed-ended questions that would be easier to implement. All questions were developed in collaboration with FHI 360 headquarters and the LINKAGES country offices in Kenya and Malawi. The questionnaire was also reviewed by key population representatives and Ministry of Health staff and piloted with three community-based organizations that were not part of our sample.

### Study setting

Both Kenya and Malawi have generalized HIV epidemics with concentrated sub-epidemics among key populations [37]. The LINKAGES program in the two countries provided a package of HIV services to key populations. The package included the following clinical services: post-exposure prophylaxis, pre-exposure prophylaxis, HIV testing services, antiretroviral therapy, sexually transmitted infection services, sexual and reproductive health services, and management of sexual violence. LINKAGES offered these interventions alongside non-clinical interventions, including empowerment and engagement services, structural interventions, and peer outreach. The program also included pre-service delivery activities, such as population mapping and size estimation, and above-service management and monitoring. This program had four management levels: 1) DICs; 2) IPs overseeing DICs; 3) LINKAGES country offices providing IPs with on-the-ground program management, capacity-building, and technical support; and 4) LINKAGES headquarters in the US providing funding, high-level program guidance, and technical assistance (S1 Fig). The links between IPs and DICs were heterogenous with some IPs overseeing one DIC only and others managing a cluster of two or more DICs. Of the 45 DICs studied (30 in Kenya and 15 in Malawi), almost every DIC had a manager who was almost always distinct from the manager of the affiliated IP—one DIC was managed by an IP manager and two DICs were managed by the same individual.

### Data collection

We administered an online facility management survey to DIC managers in Kenya and Malawi. This study on management characteristics was part of a broader scope of work, which began with a costing study of the LINKAGES program conducted in Kenya and Malawi for US Government FY 2018 and 2019 (1 October to 30 September). The collection of data on management practices was motivated by the results of the costing study, which suggested that differences in management practices across DICs might explain some unexplained variation in program costs [38, 39]. The management survey was applied from 1 November 2021 to 31 January 2022 using the Survey Monkey platform. The survey asked questions regarding the practices implemented in the DIC in FY 2019.

We contacted a total of 44 DIC managers (29 from Kenya and 15 from Malawi) with a 100% response rate. However, our sample comprises 45 observations, since two DICs in Kenya were overseen by the same manager. The survey consisted of questions on the six management domains—target setting, performance monitoring, people management, operations management, financial management, and community engagement. In addition, we also asked questions about the characteristics of the facility, personal traits of the manager, organization autonomy, and health service marketing and demand-generation practices.

### Management domain scores

We constructed six management domain scores for each DIC. The survey instrument collected data on 66 management practices (S1 Table). These 66 management practices were classified into six management domains—target setting [7], performance monitoring [18], people management [20], operations management [11], financial management [7], and community engagement [4]. Each practice was encoded dichotomously, where 1 indicated the practice was implemented in the DIC and 0 that it was not. We constructed additive linear scores for each of the six domains using the management practices in each domain. For each DIC, we summed up the *t* practices included in the *m* management domain and standardized them so that the scores ranged from 0 (lowest possible score) to 100 (highest possible score).

We measured the reliability and consistency of each management domain score using Cronbach’s alpha coefficient, which describes the inter-relatedness of the items used to calculate the score of each management domain [40, 41]. S2 Table displays these coefficients. As a general rule of thumb, an index exhibits high internal consistency if Cronbach’s alpha coefficient is higher than 0.6–0.7 [40]. Coefficients were low for target setting and operations management, due to lack of variation in some included items (e.g., all DICs said “yes”). Given that the calculation of the alpha coefficient depends on the number of items included in the index and their inter-correlation, the fact that some items were equivalent to a constant resulted in these items being excluded and not considered in the calculation of the alpha coefficient. Since these two indicators did not exhibit high internal consistency, we excluded them from this analysis.

### Data analysis

We describe the management domain scores for the DICs in the sample and the variation in scores across DICs and countries. We also describe the variation in management domains across a range of facility characteristics most of which have been found to be associated with management levels in previous studies such as a manager’s education [42, 43], the number of staff working at the DIC [10], facility maturity (the number of years since the DIC opened to 2019) [23], competition (proxied by the number of DICs within a 30 minute-driving radius providing HIV health services) [10, 44], organization size (number of staff working at the DIC) and service scale (the number of key populations reached with HIV testing services in 2019) [10–14], as well as the horizontal integration of health-services (measured by the number of HIV service types provided by a DIC in 2019). Finally, we control for the unobserved characteristics of both countries and whether DICs are overseen by an IP managing only one DIC or whether DICs belong to an IP/DIC cluster. Detailed information about the construction of these variables is found in S3 Table.

We analyzed the relationship between management domain scores and DIC characteristics using bivariate and multivariate regression models via Ordinary Least Squares (OLS). First, we regressed each management domain score against DIC characteristics, such that:

$$m_{i}=\beta_{0}+\beta_{1}c_{i}+\epsilon_{i}$$ (1)

where management domain, *m*, of DIC, *I*, is regressed against characteristic *c*.

Second, we analyzed the association between each management domain score and the set of DIC characteristics (vector *C*), controlling for country and belonging to a cluster of DICs overseen by an IP (vector *X*) such that:

$$m_{i}=\beta_{0}+\beta^{\prime }C_{i}+\alpha^{\prime }X_{i}+u_{i}$$ (2)

In both Eqs 1 and 2, *ϵ* and *u* represent the error terms of the models.

### Ethical clearance

The study was approved by the ethical review board of the National Institute of Public Health of Mexico (Number: 1554), the Kenya Medical Research Institute and the National Commission for Science, Technology, and Innovation (Protocol No. 4258), and the National Commission on Research Ethics in the Social Sciences and Humanities of Malawi (Protocol No. P/07/21/590). All DIC managers who took part in the management survey completed an electronic informed consent form. Additional information regarding the ethical, cultural, and scientific considerations specific to inclusivity in global research is included in S1 Checklist.

## Results

### DIC characteristics

**Fig 1:**
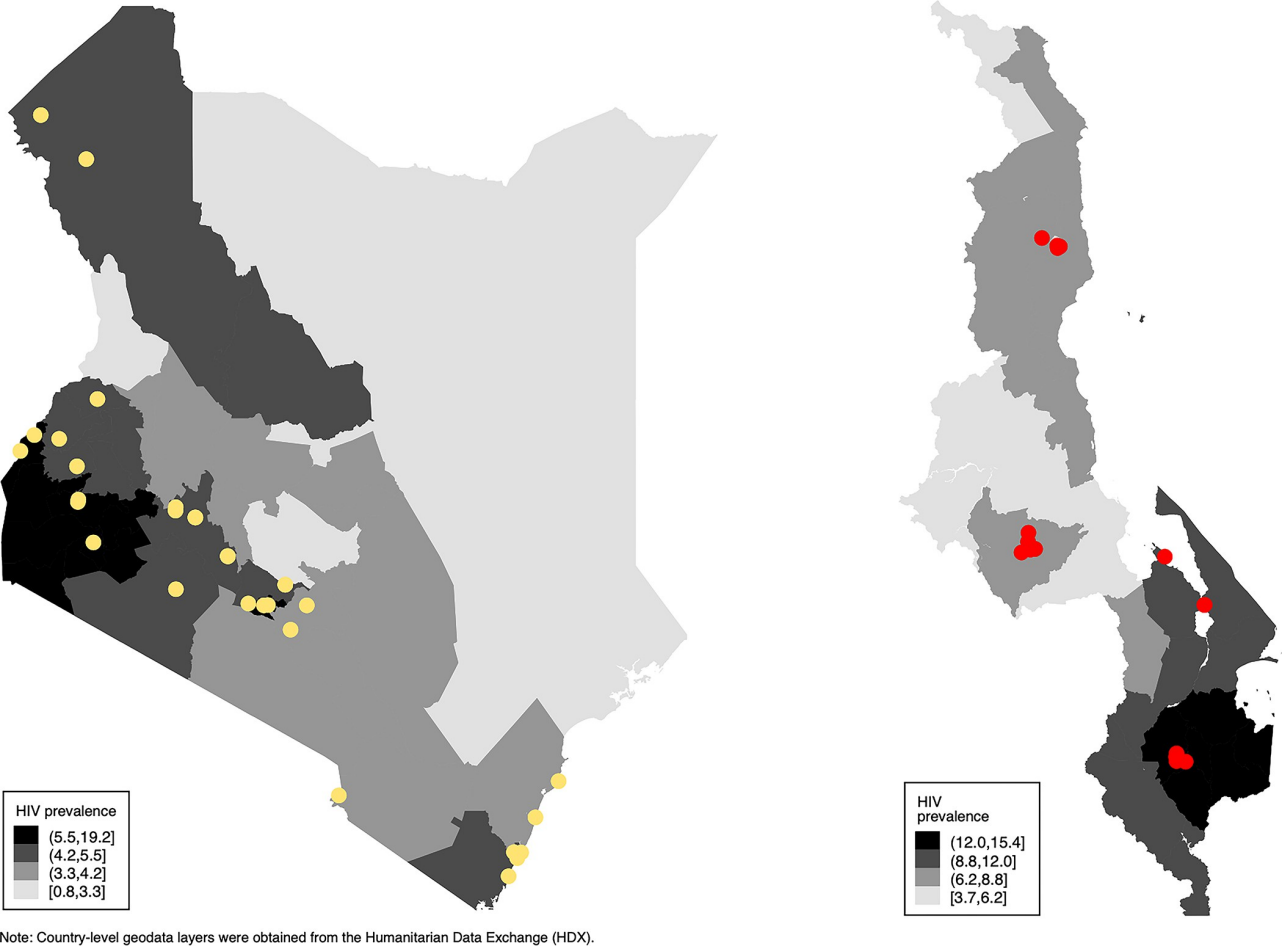
Geographic location of LINKAGES DICs in Kenya and Malawi, FY 2019. Note: Author elaboration using data on HIV prevalence from the Institute for Health Metrics and Evaluation Global Burden of Disease Study 2019. The base map of Kenya and Malawi used for this figure was obtained from the Humanitarian Data Exchange. Content from this site is licensed under a Creative Commons Attribution 4.0 International license (https://data.humdata.org/dataset/cod-ab-mwi? and https://data.humdata.org/dataset/cod-ab-ken?).

LINKAGES DICs in Kenya and Malawi were clustered in areas with higher HIV prevalence levels (Fig 1). DICs in the two countries were similar in terms of the percentage of DIC managers with university degrees, the number of staff working at DICs, and the number of HIV service types provided (Table 1). DICs in Kenya differed from those in Malawi in the following ways: they were older; they faced less competition from other DICs (i.e., there were fewer DICs clustered together within a 30-minute driving radius); they provided more services; and they were more likely to be managed by an IP administering only one DIC.

**Table 1:** Characteristics of LINKAGES DICs in Kenya and Malawi, FY 2019.

	**All sample (n=45)**			**Kenya (n=30)**			**Malawi (n=15)**		
	**Min**	**Mean**	**Max**	**Min**	**Mean**	**Max**	**Min**	**Mean**	**Max**
**Percent of DIC managers with university degree**		53.3%			50.0%			60.0%	
**Number of staff working at the DIC**	2	7	21	2	8	21	2	6	18
**Number of years from DIC opening to 2019**	1	5.2	16	1	6.4***	16	2	2.9***	4
**Number of DICs within 30 minute-driving radios providing HIV health services in 2019**	0	2.1	5	0	1.6***	4	1	2.9***	5
**Number of HIV tests provided by DIC in 2019**	16	1,595	19,647	121	2,314*	19,647	16	157*	430
**Number of HIV service types provided by DIC in 2019**	9	18	23	9	18	23	10	18	22
**Percent of DICs associated with IPs with two or more affiliated sites**		71.1%			56.7%***			100.0%***	

Notes: Statistically significant differences between the mean of both countries (+p-value < 0.10

* p-value < 0.05

** p-value < 0.01

***p-value < 0.001).

P-values were calculated with the T-student test. DICs, Drop-in centers; KPs, key populations; IPs, Implementing partners.

### Variation in management domain scores

There was substantial variation in the average management domain scores in DICs in Kenya and Malawi (Table 2 and S2 and S3 Figs). Overall, average scores were highest for performance monitoring and lowest for people management. Average scores for performance monitoring and community engagement were similar in Kenya and Malawi (Table 2) while scores for people management and financial management were higher in Kenya. Within each management domain, there was significant variation across DICs in each country. Financial management and community engagement were the two management domains with the widest ranges in scores, with some DICs having scores of 0 in these domains and others having scores of 100.

**Table 2:** Average management domains scores for LINKAGES DICs in Kenya and Malawi, FY 2019.

Management domain	All sample				Kenya Mean	Malawi Mean	Difference	p-value
	Min	Mean	Max	SD				
**Performance monitoring**	44.4	81.4	100.0	17.0	80.9	82.2	-1.3	0.812
**People management**	10.0	35.1	80.0	15.4	38.3	28.7	9.7	0.046
**Financial management**	0.0	44.8	100.0	44.3	54.3	25.7	28.6	0.04
**Community engagement**	0.0	60.0	100.0	28.4	61.7	56.7	5.0	0.584

Note: p-values were calculated with the t-student test

### Management domain score variation and DIC characteristics

**Fig 2:**
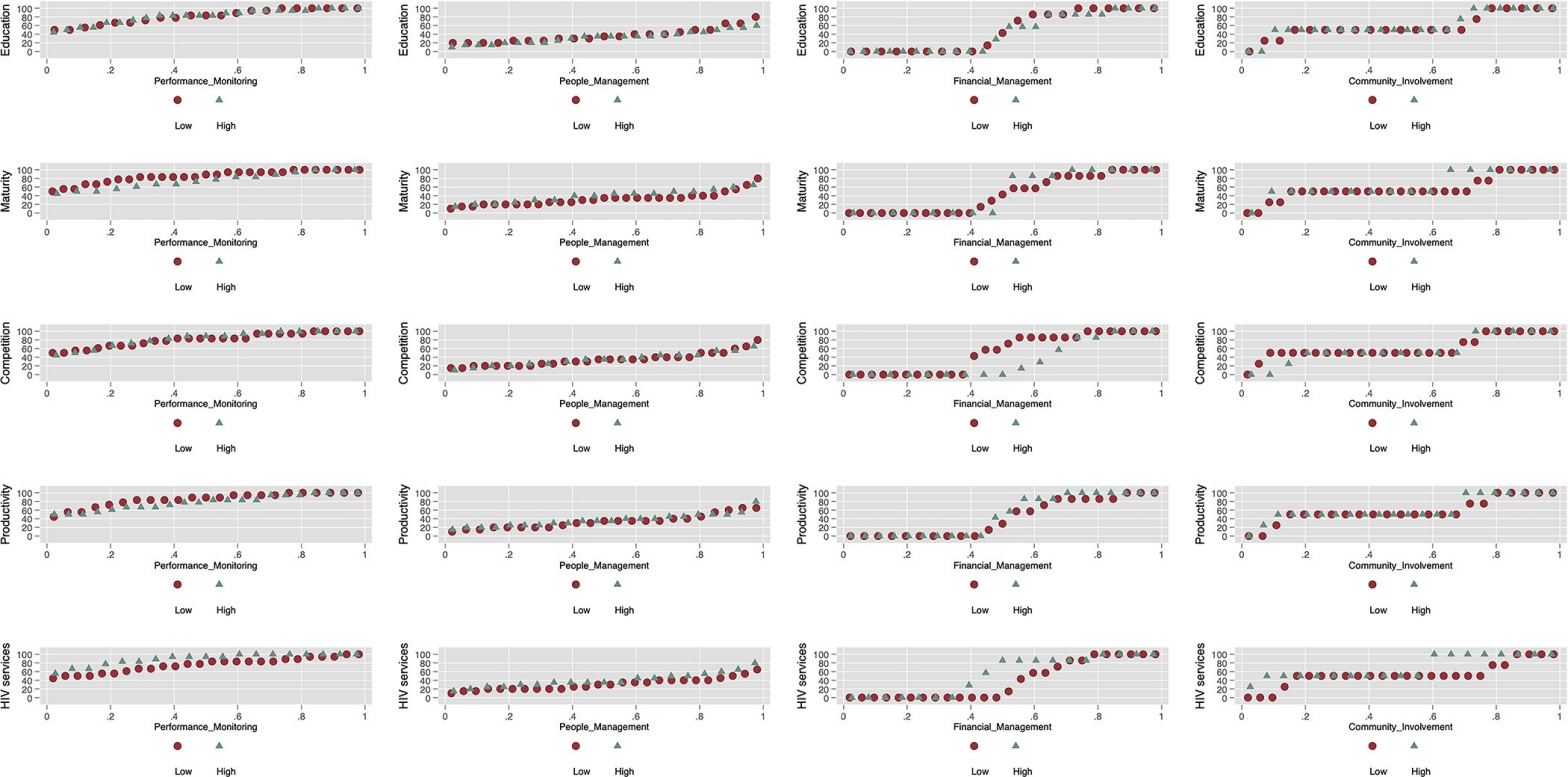
Distribution of management domains by DIC characteristics. Notes: Education: DIC managers with university degree. Maturity: number of years from DIC opening to 2019. Competition: number of DICs within 30 minute-driving radios providing HIV health services in 2019. Productivity: number of HIV testing services provided by DIC in 2019. HIV services: number of HIV service types provided by DIC in 2019. All these variables were dichotomized according to the median value. Low = below the median; High = on or above the median.

Fig 2 displays management domain score distributions by high and low levels of the different DIC characteristics highlighted in Table 1, with cut-off points defined as the medians of the DIC characteristic variables. For most management domains, there were minimal differences in scores between DICs with low and high values of DIC characteristics. There were differences in financial management scores and community engagement scores between DICs with high and low values for competition (number of DICs within a 30-minute driving radius). And financial management scores differed between DICs with high and low integration (number of HIV service types provided by the DIC) values. There were also differences in financial management scores between DICs associated with IPs with a unique DIC and DICs linked to IPs with two or more sites (S4 Table). The mean financial management score for DICs associated with IPs overseeing only one DIC was more than twice that of DICs associated with IPs managing two or more DICs. The relationships between the four management domains and DIC characteristics are further highlighted in the bivariate models shown in S5 Table. More service types provided by a DIC were associated with better management across all domains, especially for community engagement. Being linked to IPs overseeing two or more DICs was associated with worse management across all domains, especially financial management. The associations with other DIC characteristics were inconsistent across management domains.

Table 3 shows the multivariate regression results between each management domain conditional on DIC characteristics. As shown by the adjusted R-squared, there was a lot of heterogeneity in the levels of explained variation across the models. The selected DIC characteristics explained only 27% of the variation in the community engagement scores and this was the highest adjusted R-squared. Across management domains, DIC characteristics had positive and negative associations conditional on the other variables, although most of the coefficients had high levels of statistical uncertainty. Of note, each additional service type provided by a DIC was associated with increases in the community engagement score of almost five points; increases in the performance monitoring score of two points; and increases in the people management scores of over one point. Also, belonging to a cluster of DICs overseen by an IP was correlated with worse management and this was especially true for financial management.

**Table 3:** Multivariate linear regression models between facility characteristics and management domains in LINKAGES program, 2019.

DIC characteristics	Performance monitoring	People management	Financial management	Community engagement
**Percent of DIC managers with university degree**	0.66	-1.66	5.72	13.53+
	(5.18)	(4.66)	(13.47)	(7.93)
**Number of staff working at the DIC**	0.26	-0.05	-1.28	0.93
	(0.70)	(0.63)	(1.83)	(1.08)
**Number of years from DIC opening to 2019**	-1.29+	0.88	1.80	1.02
	(0.67)	(0.60)	(1.74)	(1.02)
**Number of DICs within 30 minute-driving radius providing HIV health services in 2019 **	1.88	0.84	-4.51	-1.27
	(2.19)	(1.97)	(5.69)	(3.35)
**Number of HIV tests provided by DIC in 2019 (thousands)**	-1.19	-1.28+	1.01	-1.51
	(0.78)	(0.70)	(2.03)	(1.19)
**Number of HIV service types**^#^ **provided by DIC in 2019**	2.15**	1.59*	0.33	4.90***
	(0.77)	(0.69)	(2.00)	(1.18)
**Percent of DICs associated with IPs with two or more affiliated sites**	-4.67	-10.73	-49.95*	-19.67+
	(7.30)	(6.56)	(18.98)	(11.16)
**Malawi**	-6.21	-5.94	4.39	5.21
	(7.04)	(6.33)	(18.32)	(10.77)
**_cons**	49.84**	12.45	77.31+	-31.97
	(16.84)	(15.15)	(43.81)	(25.76)
**r2_a**	.12	.14	.13	.27

Notes: _cons = intercept. Standard errors in parentheses. r2_a: Adjusted R-squared. Significantly different than zero at 99.9 (***), 99 (**), 95 (*) and 90 (+) percent confidence

^#^ Types refers to interventions, see S3 Table.

## Discussion

Management capacity is essential for organizational performance. This study analyzed the level of different management domains across community-based health facilities providing HIV services to key populations in Kenya and Malawi. We found that there was substantial variation across and within management domains and DIC characteristics explained little of this variation. We observed that providing more HIV health service types was positively correlated with performance monitoring, people management, and community engagement. We also found that belonging to a cluster of DICs overseen by an IP was correlated with lower management capacity.

Our findings on the variation in management domain scores across DICs are consistent with previous work showing the heterogeneity in management practices across organizations [10, 11, 13, 14]. The management domain scores in this sample of community-based health facilities suggest that though management capacity varied across DICs, in some of these organizations, management capacity was high. Management capacity varied by domain, with overall performance monitoring and community engagement scores higher than financial management and people management scores. However, even for those management domains with lower scores—financial management and people management—the scores for some DICs were high. These findings contrast with the results of previous studies of community-based organizations providing HIV services, which all found low levels of management capacity across organizations [21–23]. Part of this difference in results may be attributable to the unique structure of the LINKAGES program where DICs were overseen and supported by IPs, which in turn received oversight and assistance from country offices and headquarters as well as the Ministry of Health. This supposition would appear to be supported by our finding that management scores, especially financial management scores, were lower in DICs that were overseen by an IP that managed more than one DIC. Our findings suggest that community-based health facilities can have high levels of management in a context where they are provided with adequate levels of above-facility support and oversight—even if they deliver complex services, may rely heavily on temporary workers and community volunteers, and face significant financial constraints.

Previous studies conducted in high-income countries have found that the number of facility employees [10] and competition faced [11, 44] by a facility were positively associated with management. In contrast, we did not find consistent evidence of a positive association between management domain scores and the number of staff and competition. On competition, the presence of other community-based health facilities providing similar services within a 30-minute drive radius may not necessarily mean that facilities compete to serve a higher number of key populations. Lack of planning and coordination between internationally funded non-governmental organizations and publicly funded agencies has led to duplication and competitive practices [45]. However, in Kenya, non-governmental organizations funded by PEPFAR and those funded by the Global Fund collaborated to provide services to key populations [46]. Granular data about key population HIV prevalence and environmental factors such as urbanity, population density, etc., would be needed to better explore the association between competition and management. This is something that future studies should consider.

Previous studies have also shown that a facility manager’s training and formal education were positively associated with management scores [10, 42, 47] but we found only limited evidence of a positive relationship between a DIC manager’s education in only one domain—community engagement. This association between a DIC manager’s education and community engagement is difficult to interpret without qualitative research and additional contextual information to understand how social, economic, and other differences interact with educational backgrounds. One potential explanation might be that the structured aspects of community engagement might be less understood or less compelling to managers with lower levels of education, given that informal community engagement is likely a regular practice in DICs. Generally, our finding on the weak association between a manager’s education and management suggests that community-based health facilities can have high levels of management in a context where they are provided with adequate levels of above-facility support and oversight—irrespective of the education level of the facility manager. It is probable that whether a manager is a member of the population served and the empathy and knowledge associated with this status also play a role in the management of such organizations. Further research—both quantitative and qualitative—is needed to explore the association between a manager’s education and management in community-based organizations working in HIV services and in other health areas.

Our results that providing more HIV health service types was positively correlated with better management, especially for community engagement and performance monitoring is noteworthy. Offering a wider range of HIV services at a DIC likely increases operational complexity. This encompasses the greater logistical challenges of ensuring that more service types are delivered effectively. It includes addressing a wider spectrum of client needs, which can vary significantly from one service type to another. It also involves managing a broader range of inputs, from drugs and medical supplies to human resources. More horizontal integration may incentivize or even require facilities to adopt better management practices to cope with the increased complexity of providing more service types—especially to increase performance monitoring practices and expand community relationships. It is also possible, however, that more effective management in DICs results in an expanded range of service offerings to better meet community needs.

Finally, our result concerning worse financial management in DICs belonging to a cluster of DICs might be linked to lack of peer effects in the implementation of financial procedures. This suggests that IPs carried out a lot of the financial management. This centralization of financial management may have been a lost opportunity for local non-governmental organizations to improve their administrative capacity and financial competence.

Though the management practices we studied were based on previous work and validated by staff overseeing and supporting DICs, the following limitations should be kept in mind when considering this work. Our study’s reliance on closed-ended questions for assessing management practices may not provide as thorough an understanding as the original, in-depth qualitative methodology of the WMS [36]. However, several studies implemented in primary health care facilities in low-resource settings using closed-ended questions derived from the WMS have highlighted the value of this more efficient method [16, 18]. It is also likely that including questions on facility and manager characteristics added to the complexity of the questionnaire, but previous work has shown the feasibility of this approach in other types of facilities [16, 18]. We note that two of the management domain scores constructed—target setting and operations management—had low levels of internal consistency and were excluded from this analysis. Identifying the adequate management practices associated with the target setting and operations management domains in community-based health facilities requires further research. This was a cross-sectional study and as such does not account for the dynamic nature of management practices which tend to evolve positively over time [48, 49], although we accounted for DIC maturity in our analysis. Our analyses also rely on a small sample, and this implies that findings from our multivariate models should be viewed with caution. Another limitation is that managers were asked about the management practices they were responsible for, and they may have had the incentive to represent themselves as positively as possible. Also, because of practical constraints, data on management practices in the DICs were asked two years after their implementation and recall bias might introduce errors in the calculation of the management scores. Notwithstanding these limitations, this study contributes to the ongoing literature about the role of management practices across organizations that provide health services.

## Conclusions

This paper contributes to the growing literature on management practices in health service facilities in resource-limited settings. Though we found a lot of heterogeneity in management practices across facilities, some community-based health facilities providing HIV services to key populations had high levels of management. These organizations operated in a particular context where they were provided with significant levels of above-facility support and oversight. The variation in management scores across DICs also suggests varying support and oversight needs. Importantly, our results indicate that community-based organizations that deliver complex services and face significant financial constraints can have strong management practices. The potential for deriving policy recommendations from a cross-sectional study is inherently limited. However, our findings suggest the effectiveness of structured support and oversight for CBO managers, even managers with less education. This implies that high-quality management in CBOs is achievable with the appropriate systems, irrespective of the educational backgrounds of the managers. The intensive nature of the support in the LINKAGES program raises questions about the necessary intensity of the oversight and support measures. Future research should explore the required intensity and efficacy of lighter approaches on CBO management levels including on the job management training programs and ongoing mentorship. Regardless of the intensity, a delicate balance should be reached between capacity building and structured oversight and support, while also addressing the distinct needs of each CBO and avoiding excessive bureaucracy and unnecessary stress on CBO managers.

## Supporting information

S1 Checklist Inclusivity in global research. (DOCX)

S1 Fig LINKAGES program structure (implementation levels and sample size). Notes: LINKAGES, Linkages Across the Continuum of HIV Services for Key Populations Affected by HIV; IP, Implementing Partner; DIC, Drop-in-center; n, number of offices/organizations/facilities.
(TIFF)

S2 Fig Average management domain scores for LINKAGES DICs in Kenya and Malawi, 2019. Notes: Histograms show the distribution of the scores for each management domain. Number of observations: 30 Kenya, 15 Malawi.
(TIF)

S3 Fig Heterogeneity of management domains across countries. Notes: Bars in light blue represent Kenya, whereas those in dark navy depict Malawi. Number of observations: 30 Kenya, 15 Malawi.
(TIF)

S1 Table List of management practices for each management domain. Notes: All items coded as 1 = Yes, 0 = No.
(DOCX)

S2 Table Cronbach’s alpha coefficients for each management domain score. Notes: Average scores by management domains. Error bars represent confidence intervals at 95% of confidence. Number of observations: 45 DICs in Kenya and Malawi, 30 DICs in Kenya, 15 DICS in Malawi.
(DOCX)

S3 Table Description of DIC characteristics. (DOCX)

S4 Table Average scores for management domains in DICs by type of IP. Notes: P-values were calculated with the T-student test.
(DOCX)

S5 Table Bivariate linear regression models. Notes: Standard errors in parentheses. r2_a: Adjusted R-squared. Significantly different than zero at 99.9 (***), 99 (**), 95 (*) and 90 (+) percent confidence. ^#^ Types refers to interventions, see S3 Table.
(DOCX)

## References

[1] Joint United Nations Programme on HIV/AIDS. In Danger: UNAIDS Global AIDS Update 2022. Geneva: Joint United Nations Programme on HIV/AIDS, 2022. 12349391

[2] Barr D, Garnett GP, Mayer KH, Morrison M. Key populations are the future of the African HIV/AIDS pandemic. J Int AIDS Soc. 2021;24 Suppl 3:e25750. Epub 2021/07/01. doi: 10.1002/jia2.25750 ; PubMed Central PMCID: PMC8242978. 34189865 PMC8242978

[3] Garnett GP. Reductions in HIV incidence are likely to increase the importance of key population programmes for HIV control in sub-Saharan Africa. J Int AIDS Soc. 2021;24 Suppl 3:e25727. Epub 2021/07/01. doi: 10.1002/jia2.25727 ; PubMed Central PMCID: PMC8242973. 34189844 PMC8242973

[4] International AIDS Society. Differentiated service delivery: a decision framework for differentiated antiretroviral therapy for key populations. Amsterdam: International AIDS Society, 2018.

[5] World Health Organization. Consolidated Guidelines on HIV Prevention, Diagnosis, Treatment and Care for Key Populations. Geneva: World Health Organization, 2016. 27559558

[6] Bloom N, Reenen J. Human resource management and productivity. In: Card D, Ashenfelter O, editors. Handbook of labor economics. Volume 4, Part B. Amsterdam, New York, Oxford: Elsevier Science, North-Holland; 2011. p. 1697–769.

[7] Bloom N, Van Reenen J. Measuring and explaining management practices across firms and countries. Q J Econ. 2007;122(4):1351–408. doi: 10.1162/qjec.2007.122.4.1351

[8] Bradley EH, Taylor LA, Cuellar CJ. Management Matters: A Leverage Point for Health Systems Strengthening in Global Health. Int J Health Policy Manag. 2015;4(7):411–5. Epub 2015/07/21. doi: 10.15171/ijhpm.2015.101 ; PubMed Central PMCID: PMC4493581. 26188805 PMC4493581

[9] Lega F, Prenestini A, Spurgeon P. Is management essential to improving the performance and sustainability of health care systems and organizations? A systematic review and a roadmap for future studies. Value Health. 2013;16(1 Suppl):S46-51. Epub 2013/01/18. doi: 10.1016/j.jval.2012.10.004 . 23317645

[10] Bloom N, Sadun R, Van Reenen J. Does management matter in healthcare? London: LSE Working Paper, 2013.

[11] Dorgan S, Layton D, Bloom N, Homkes R, Sadun R, Van Reenen J. Management in healthcare: why good practice really matters. London: McKinsey & Company, 2010.

[12] McConnell KJ, Chang AM, Maddox TM, Wholey DR, Lindrooth RC. An exploration of management practices in hospitals. Healthc (Amst). 2014;2(2):121–9. Epub 2015/08/08. doi: 10.1016/j.hjdsi.2013.12.014 . 26250380

[13] McConnell KJ, Lindrooth RC, Wholey DR, Maddox TM, Bloom N. Management practices and the quality of care in cardiac units. JAMA Intern Med. 2013;173(8):684–92. Epub 2013/04/05. doi: 10.1001/jamainternmed.2013.3577 . 23552986

[14] McConnell KJ, Lindrooth RC, Wholey DR, Maddox TM, Bloom N. Modern management practices and hospital admissions. Health Econ. 2016;25(4):470–85. Epub 2015/02/26. doi: 10.1002/hec.3171 . 25712429

[15] Tsai TC, Jha AK, Gawande AA, Huckman RS, Bloom N, Sadun R. Hospital board and management practices are strongly related to hospital performance on clinical quality metrics. Health Aff (Millwood). 2015;34(8):1304–11. Epub 2015/08/05. doi: 10.1377/hlthaff.2014.1282 . 26240243

[16] Kim JH, Bell GA, Bitton A, Desai EV, Hirschhorn LR, Makumbi F, et al. Health facility management and primary health care performance in Uganda. BMC Health Serv Res. 2022;22(1):275. Epub 2022/03/03. doi: 10.1186/s12913-022-07674-3 ; PubMed Central PMCID: PMC8886189. 35232451 PMC8886189

[17] Mabuchi S, Alonge O, Tsugawa Y, Bennett S. An investigation of the relationship between the performance and management practices of health facilities under a performance-based financing scheme in Nigeria. Health Policy Plan. 2022;37(7):836–48. Epub 2022/05/18. doi: 10.1093/heapol/czac040 . 35579285

[18] Macarayan EK, Ratcliffe HL, Otupiri E, Hirschhorn LR, Miller K, Lipsitz SR, et al. Facility management associated with improved primary health care outcomes in Ghana. PLoS One. 2019;14(7):e0218662. Epub 2019/07/03. doi: 10.1371/journal.pone.0218662 ; PubMed Central PMCID: PMC6605853 31265454 PMC6605853

[19] Sosa-Rubi SG, Bautista-Arredondo S, Chivardi-Moreno C, Contreras-Loya D, La Hera-Fuentes G, Opuni M. Efficiency, quality, and management practices in health facilities providing outpatient HIV services in Kenya, Nigeria, Rwanda, South Africa and Zambia. Health Care Manag Sci. 2021;24(1):41–54. Epub 2021/02/06. doi: 10.1007/s10729-020-09541-1 . 33544323

[20] Salas-Ortiz A, La Hera-Fuentes G, Nance N, Sosa-Rubi SG, Bautista-Arredondo S. The relationship between management practices and the efficiency and quality of voluntary medical male circumcision services in four African countries. PLoS One. 2019;14(10):e0222180. Epub 2019/10/04. doi: 10.1371/journal.pone.0222180 ; PubMed Central PMCID: PMC6776351. 31581192 PMC6776351

[21] Kegeles SM, Rebchook G, Pollack L, Huebner D, Tebbetts S, Hamiga J, et al. An intervention to help community-based organizations implement an evidence-based HIV prevention intervention: the Mpowerment Project technology exchange system. Am J Community Psychol. 2012;49(1–2):182–98. Epub 2011/06/22. doi: 10.1007/s10464-011-9451-0 . 21691911

[22] Mayberry RM, Daniels P, Akintobi TH, Yancey EM, Berry J, Clark N. Community-based organizations’ capacity to plan, implement, and evaluate success. J Community Health. 2008;33(5):285–92. Epub 2008/05/27. doi: 10.1007/s10900-008-9102-z ; PubMed Central PMCID: PMC3782989. 18500451 PMC3782989

[23] Akeju D, Nance N, Salas-Ortiz A, Fakunmoju A, Ezirim I, Oluwayinka AG, et al. Management practices in community-based HIV prevention organizations in Nigeria. BMC Health Serv Res. 2021;21(1):489. Epub 2021/05/24. doi: 10.1186/s12913-021-06494-1 ; PubMed Central PMCID: PMC8141130. 34022857 PMC8141130

[24] Beattie TS, Bhattacharjee P, Suresh M, Isac S, Ramesh BM, Moses S. Personal, interpersonal and structural challenges to accessing HIV testing, treatment and care services among female sex workers, men who have sex with men and transgenders in Karnataka state, South India. J Epidemiol Community Health. 2012;66 Suppl 2:ii42-8. Epub 2012/04/13. doi: 10.1136/jech-2011-200475 . 22495772

[25] Laga M, Vuylsteke B. Evaluating AVAHAN’s design, implementation and impact: lessons learned for the HIV Prevention Community. BMC Public Health. 2011;11 Suppl 6:S16. Epub 2012/03/02. doi: 10.1186/1471-2458-11-S6-S16 ; PubMed Central PMCID: PMC3287554. 22376320 PMC3287554

[26] Vassall A, Chandrashekar S, Pickles M, Beattie TS, Shetty G, Bhattacharjee P, et al. Community mobilisation and empowerment interventions as part of HIV prevention for female sex workers in Southern India: a cost-effectiveness analysis. PLoS One. 2014;9(10):e110562. Epub 2014/10/22. doi: 10.1371/journal.pone.0110562 ; PubMed Central PMCID: PMC4204894. 25333501 PMC4204894

[27] Herce ME, Miller WM, Bula A, Edwards JK, Sapalalo P, Lancaster KE, et al. Achieving the first 90 for key populations in sub-Saharan Africa through venue-based outreach: challenges and opportunities for HIV prevention based on PLACE study findings from Malawi and Angola. J Int AIDS Soc. 2018;21 Suppl 5:e25132. Epub 2018/07/24. doi: 10.1002/jia2.25132 ; PubMed Central PMCID: PMC6055127. 30033589 PMC6055127

[28] Joint United Nations Programme on HIV/AIDS. UNAIDS guidance for partnerships with civil society, including people living with HIV and key populations. Geneva: Joint United Nations Programme on HIV/AIDS, 2011.

[29] Weir SS, Figueroa JP, Scott M, Byfield L, Jones Cooper C, Hobbs MC. Reaching key populations through key venues: Insights from the Jamaica HIV Prevention Program. PLoS One. 2018;13(11):e0206962. Epub 2018/11/27. doi: 10.1371/journal.pone.0206962 ; PubMed Central PMCID: PMC6261031. 30475802 PMC6261031

[30] DiCarlo MC, Dallabetta GA, Akolo C, Bautista-Arredondo S, Digolo HV, Fonner VA, et al. Adequate funding of comprehensive community-based programs for key populations needed now more than ever to reach and sustain HIV targets. J Int AIDS Soc. 2022;25(7):e25967. Epub 2022/07/27. doi: 10.1002/jia2.25967 ; PubMed Central PMCID: PMC9318644. 35880969 PMC9318644

[31] Benotsch EG, Stevenson LY, Sitzler CA, Kelly JA, Makhaye G, Mathey ED, et al. HIV prevention in Africa: programs and populations served by non-governmental organizations. J Community Health. 2004;29(4):319–36. Epub 2004/06/10. doi: 10.1023/b:johe.0000025329.10411.2a . 15186017

[32] Bloom N, Lemos R, Sadun R, Scur D, Van Reenen J. The new empirical economics of management. J Eur Econ Assoc. 2014;12:835–76. doi: 10.1111/jeea.12094

[33] Bloom N, Sadun R, Van Reenen J. Does management really work? Harv Bus Rev. 2012;90(11):77–80, 2, 148. Epub 2012/11/20. . 23155999

[34] Scur D, Sadun R, Van Reenen J, Lemos R, Bloom N. The World Management Survey at 18: lessons and the way forward. Oxford Rev Econ Policy 2021;37(2):231–58. doi: 10.1093/oxrep/grab009

[35] Mabuchi S, Alonge O, Tsugawa Y, Bennett S. Measuring management practices in primary health care facilities—development and validation of management practices scorecard in Nigeria. Glob Health Action. 2020;13(1):1763078. Epub 2020/06/09. doi: 10.1080/16549716.2020.1763078 ; PubMed Central PMCID: PMC7448912. 32508273 PMC7448912

[36] Bloom N, Lemos R, Sadun R, Scur D, Van Reenen J. International data on measuring management practices. Am Econ Rev. 2016;106(5):152–6. doi: 10.1257/aer.p20161058

[37] Tanser F, de Oliveira T, Maheu-Giroux M, Barnighausen T. Concentrated HIV subepidemics in generalized epidemic settings. Curr Opin HIV AIDS. 2014;9(2):115–25. Epub 2013/12/21. doi: 10.1097/COH.0000000000000034 ; PubMed Central PMCID: PMC4228373. 24356328 PMC4228373

[38] Opuni M, Figueroa JL, Sanchez-Morales JE, Salas-Ortiz A, Ochoa-Sanchez LE, Morales-Vazquez M, et al. The Cost of Providing Comprehensive HIV Services to Key Populations: An Analysis of the LINKAGES Program in Kenya and Malawi. Glob Health Sci Pract. 2023;11(3). Epub 20230621. doi: 10.9745/GHSP-D-22-00538 ; PubMed Central PMCID: PMC10285728. 37348941 PMC10285728

[39] Opuni M, Sanchez-Morales JE, Figueroa JL, Salas-Ortiz A, Banda LM, Olawo A, et al. Estimating the cost of HIV services for key populations provided by the LINKAGES program in Kenya and Malawi. BMC Health Serv Res. 2023;23(1):337. Epub 20230404. doi: 10.1186/s12913-023-09279-w ; PubMed Central PMCID: PMC10071702. 37016402 PMC10071702

[40] Tavakol M, Dennick R. Making sense of Cronbach’s alpha. Int J Med Educ 2011;2:53–5. doi: 10.5116/ijme.4dfb.8dfd 28029643 PMC4205511

[41] UCLA Statistical Consulting Group. What does Cronbach’s Alpha Mean? SPSS FAQ 2021. Available from: https://stats.oarc.ucla.edu/spss/faq/what-does-cronbachs-alpha-mean/#:~:text=Cronbach’s.

[42] Goodall AH. Physician-leaders and hospital performance: is there an association? Soc Sci Med. 2011;73(4):535–9. Epub 2011/08/02. doi: 10.1016/j.socscimed.2011.06.025 . 21802184

[43] Janke K, Propper C, Sadun R. The impact of CEOs in the public sector: evidence from the English NHS. Cambridge: National Bureau of Economic Research, 2019.

[44] Bloom N, Propper C, Seiler S, Van Reenen J. The impact of competition on management quality: evidence from public hospitals. Rev Econ Stud. 2015;82(2):457–89. doi: 10.1093/restud/rdu045

[45] Biesma RG, Brugha R, Harmer A, Walsh A, Spicer N, Walt G. The effects of global health initiatives on country health systems: a review of the evidence from HIV/AIDS control. Health Policy Plan. 2009;24(4):239–52. Epub 2009/06/06. doi: 10.1093/heapol/czp025 ; PubMed Central PMCID: PMC2699244. 19491291 PMC2699244

[46] National AIDS and STI Control Programme. Key population mapping and size estimation in selected counties in Kenya: phase 1 key findings. Nairobi: National AIDS and STI Control Programme, 2019.

[47] Mascia D, Piconi I. Career histories and managerial performance of health care chief executive officers: an empirical study in the Italian National Health Service. Health Care Manage Rev. 2013;38(1):71–80. Epub 2011/12/14. doi: 10.1097/HMR.0b013e31823dc85b . 22157466

[48] Bloom N, Brynjolfsson E, Foster L, Jarmin R, Patnaik M, Saporta-Eksten I, et al. What drives differences in management practices. Am Econ Rev. 2019;109(5):1648–83. doi: 10.1257/aer.20170491

[49] Bloom N, Van Reenen J. Why do management practices differ across firms and countries? J Econ Perspect. 2010;24(1):203–24. doi: 10.1257/jep.24.1.203

