# Supplementary figures and images for: Management practices in facilities providing HIV services to key populations in Kenya and Malawi: A descriptive analysis of management in community-based organizations
Source: PLOS Glob Public Health. 2024 Mar 20;4(3):e0002813. doi: 10.1371/journal.pgph.0002813 (PMC10954182; doi:10.1371/journal.pgph.0002813)

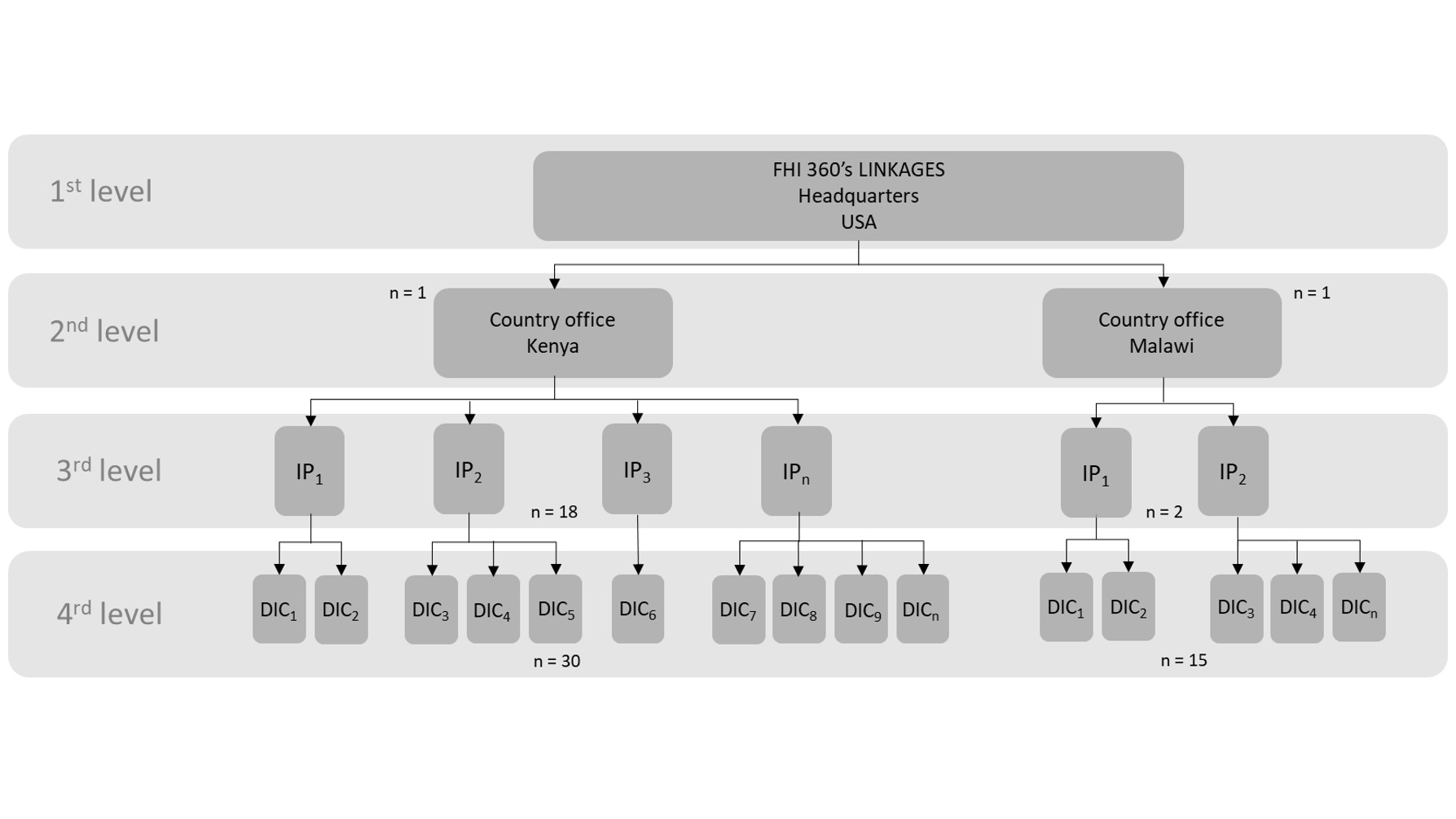

Supplement: S1 Fig — Notes: LINKAGES, Linkages Across the Continuum of HIV Services for Key Populations Affected by HIV; IP, Implementing Partner; DIC, Drop-in-center; n, number of offices/organizations/facilities. (TIFF) [file pgph.0002813.s002.tiff]

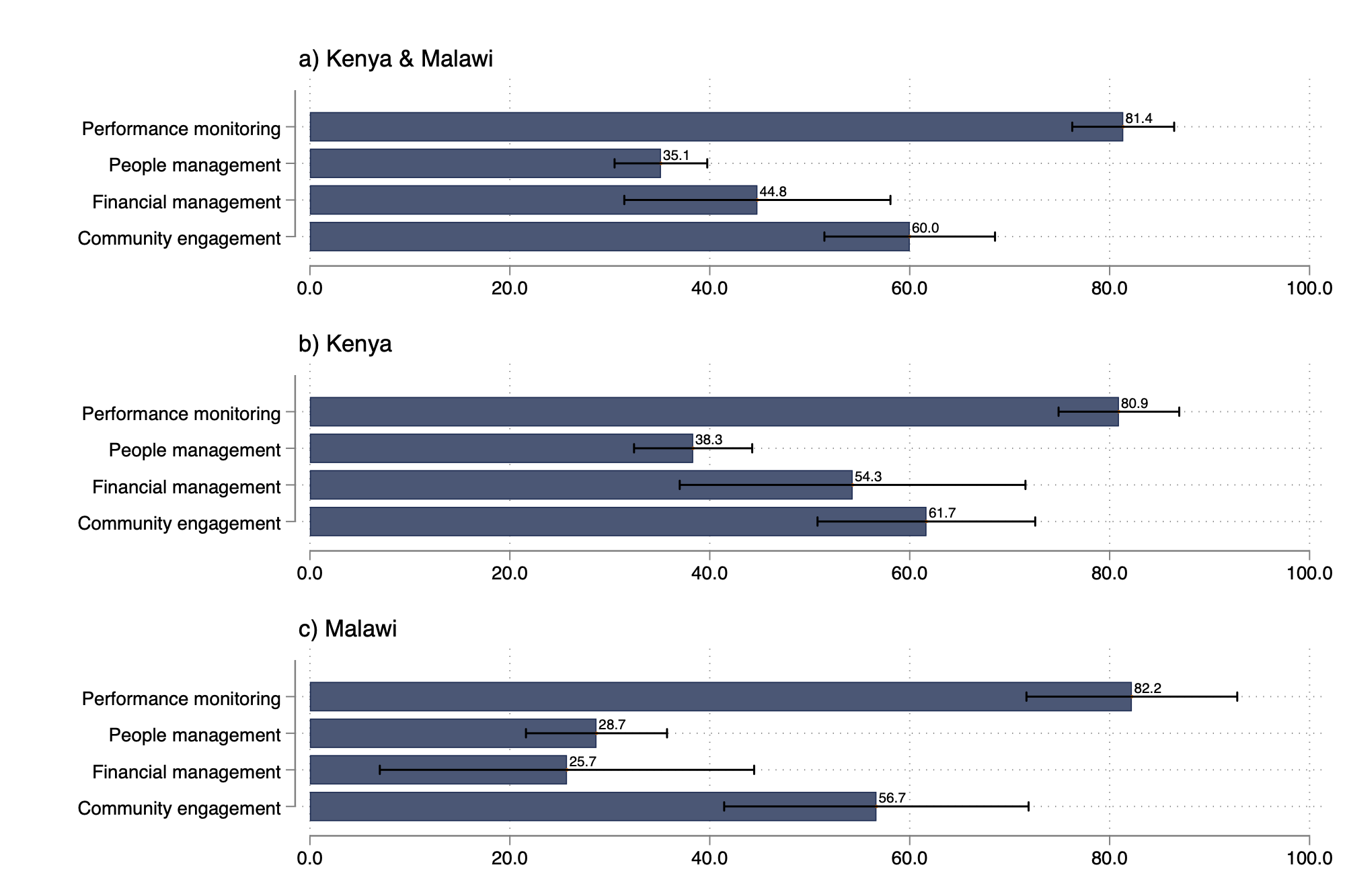

Supplement: S2 Fig — Notes: Histograms show the distribution of the scores for each management domain. Number of observations: 30 Kenya, 15 Malawi. (TIF) [file pgph.0002813.s003.tif]

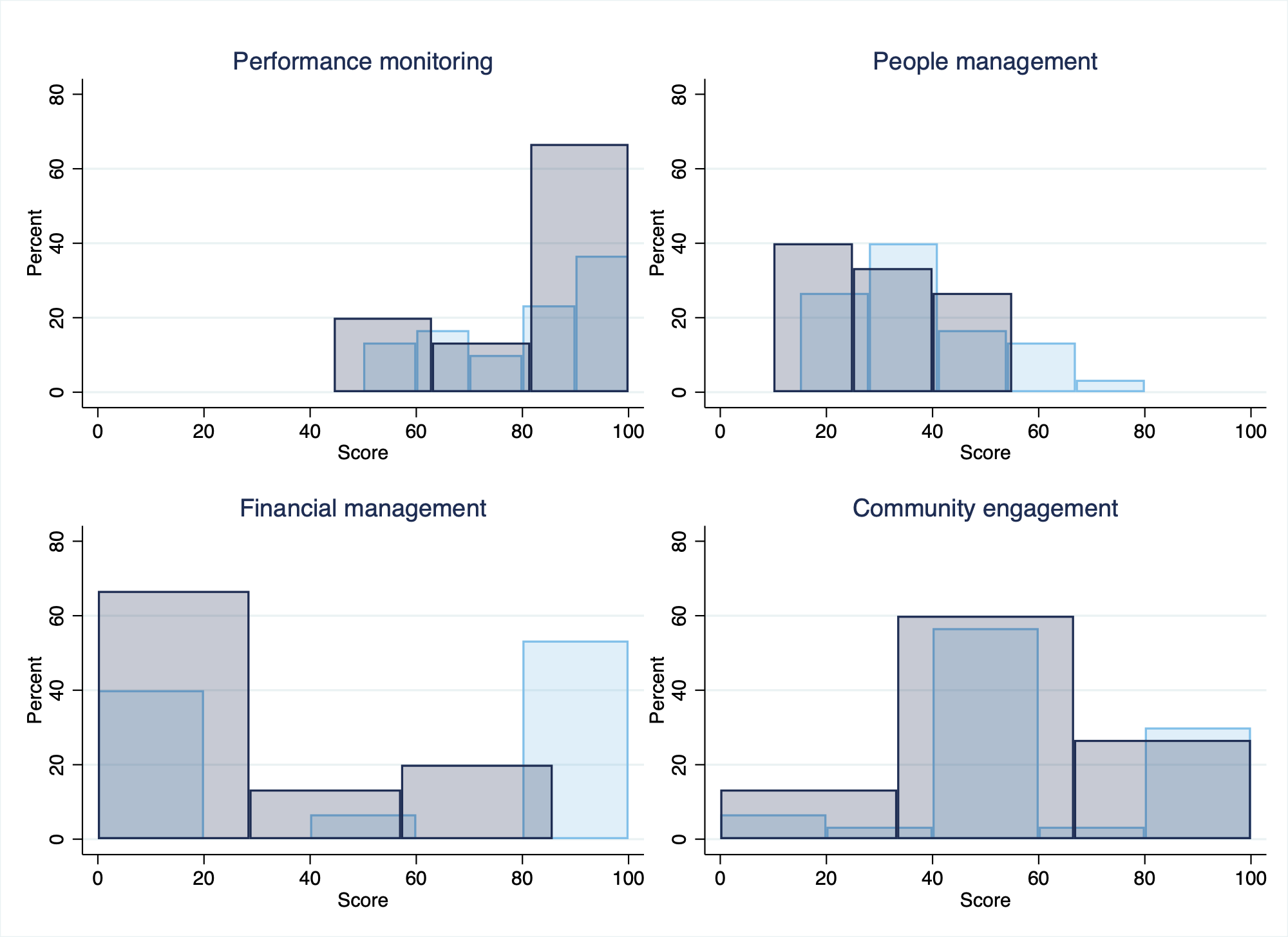

Supplement: S3 Fig — Notes: Bars in light blue represent Kenya, whereas those in dark navy depict Malawi. Number of observations: 30 Kenya, 15 Malawi. (TIF) [file pgph.0002813.s004.tif]
