# Supplementary material for: Management practices in facilities providing HIV services to key populations in Kenya and Malawi: A descriptive analysis of management in community-based organizations
Source: PLOS Glob Public Health. 2024 Mar 20;4(3):e0002813. doi: 10.1371/journal.pgph.0002813 (PMC10954182; doi:10.1371/journal.pgph.0002813)
Supplement: S1 Table — Notes: All items coded as 1 = Yes, 0 = No. (DOCX) [file pgph.0002813.s005.docx]

| **Target setting** | **Activities carried out at the DIC during 2018- 2019** |
| --- | --- |
|  | 1. DIC-specific goals were set every year, for example number of individuals reached or testing rates |
|  | 2. The DIC had goals related to the spending plan (budget). |
|  | 3. The DIC set goals related to community involvement. |
|  | 4. The DIC set goals related to marketing and demand generation. |
|  | 5. The DIC set goals for individual staff members. |
|  | 6. The DIC set goals at the team level. |
|  | 7. A timeline was made and updated to reach the staff goals and targets. |
| **Performance**  **monitoring** | **Activities carried out at the DIC during 2018- 2019** |
|  | 1. The DIC was required to inform external entities about facility performance. |
|  | 2. The DIC was required to inform external entities about staff performance. |
|  | 3. The DIC informed external entities about their performance on meeting supply targets |
|  | 4. The DIC informed external entities about their performance on meeting budgets and expenditures |
|  | 5. The DIC, procedures were sent/shown to an external entity. |
|  | 6. The DIC organized internal meetings to report DIC performance. |
|  | 7. The in-country office supervised the frequency and content of meetings held at the DIC |
|  | 8. The DIC was required to send evidence of meetings and agreements related to service provision |
|  | **External entity evaluation** |
|  | 1. External entity evaluate the DIC in terms of: Stock of supplies |
|  | 2. External entity evaluate the DIC in terms of: Staff performance |
|  | 3. External entity evaluate the DIC in terms of: Budget and expenditures |
|  | 4. External entity evaluate the DIC in terms of: Quality of care |
|  | 5. External entity evaluate the DIC in terms of: DIC layout |
|  | **Periodic internal review evaluation** |
|  | 1. The DIC engage in periodic internal review evaluation of: Stock of supplies |
|  | 2. The DIC engage in periodic internal review evaluation of: Staff performance |
|  | 3. The DIC engage in periodic internal review evaluation of: Budget and expenditures |
|  | 4. The DIC engage in periodic internal review evaluation of: Quality of care |
|  | 5. The DIC engage in periodic internal review evaluation of: DIC layout |
| **People**  **management** | **Structures or activities that were normally presented or carried out at the DIC during 2018/2019** |
|  | 1. The DIC evaluates the performance of its operational staff. |
|  | 2. Staff members receive incentives or rewards to recognize their performance |
|  | 3. Staff members receive sanctions for poor performance. |
|  | 4. Staff that is not engaged or is partially engaged, receives the full salary at the month end |
|  | **Types of incentives or rewards implemented among staff during 2018/2019** |
|  | 1. Time off |
|  | 2. Verbal recognition |
|  | 3. Written recognition / certificates |
|  | 4. Monetary bonuses |
|  | 5. Subsidized trainings/courses |
|  | 6. Preferred schedule |
|  | 7. Commodities (e.g., food basket) |
|  | **Types of incentives sanctions or rewards were implemented among staff during 2018/2019** |
|  | 1. Verbal warning |
|  | 2. Written warning |
|  | 3. Forced leave or relocation |
|  | 4. Pay reduction |
|  | 5. Become ineligible for monetary bonuses (e.g., per diem, punctuality bonus) |
|  | 6. Less desirable schedule |
|  | **Training** |
|  | 1. DIC manager or any of the employees of the facility attended any training |
|  | 2. The facility have training plans for clinical staff |
|  | 3. During 2018-2019, the DIC manager was trained in topics related to general management |
| **Operations**  **management** | **Structures or activities were normally present / carried out at the DIC during 2018-2019** |
|  | 1. The DIC has an established schedule to perform organization-related activities, such as sorting, labelling, and filing documents (e.g., patient records, MoH registers) |
|  | 2. The DIC has a dedicated space to store lab inputs (e.g., Rapid Diagnostic Tests (RDTs), lab reagents) |
|  | 3. The DIC has a dedicated space to store drugs |
|  | 4. Medical supplies are stored and organized, as soon as they arrive |
|  | 5. There is a documented process that describes how to manage drug stock, RDTs and other lab inputs |
|  | 6. The DIC does not feel crowded to walk by |
|  | 7. The DIC usually operates in special schedules, to reach key populations (e.g., night shifts, weekends) |
|  | 8. When the workload is heavy, the DIC employs locum nurses from other health facilities (e.g., government hospitals) |
|  | 9. Procedures for staff are documented in a manual and easily accessible for consultation (SOPs) |
|  | 10. Procedures are posted on the walls or blackboards of the DIC |
|  | 11. Reminders are posted in a special place of the DIC (e.g., color-coded biohazard disposal reminders) |
| **Financial**  **management** | **Structures or activities were normally present / carried out at the DIC during 2018-2019** |
|  | 1. The DIC handle financial resources directly, e.g., revenue for providing health services, direct donations for purchasing inputs/paying utilities |
|  | 2. The DIC provides a LINKAGES financial report every month |
|  | 3. The DIC does a financial audit for itself every year on total facilities revenues and expenditures |
|  | 4. Someone outside of the DIC (externals) come to do a financial audit every year on total DIC revenues and expenditures |
|  | 5. The DIC elaborates spending plans (budget) each year |
|  | 6. All payments are done through the bank / mobile money |
|  | 7. The DIC has a dedicated bank account to manage DIC expenses |
| **Community**  **engagement** | 1. During 2018-2019, the DIC had a DIC committee / governing board |
|  | 2. During 2018-2019, members of the community involved in the DIC committee / governing board |
|  | 3. During 2018-2019, members of the community participated in the budget decision making process |
|  | 4. During 2018-2019, members of the community participated in expenditures decisions? |
