# Supplementary material for: Management practices in facilities providing HIV services to key populations in Kenya and Malawi: A descriptive analysis of management in community-based organizations
Source: PLOS Glob Public Health. 2024 Mar 20;4(3):e0002813. doi: 10.1371/journal.pgph.0002813 (PMC10954182; doi:10.1371/journal.pgph.0002813)
Supplement: S2 Table — Notes: Average scores by management domains. Error bars represent confidence intervals at 95% of confidence. Number of observations: 45 DICs in Kenya and Malawi, 30 DICs in Kenya, 15 DICS in Malawi. (DOCX) [file pgph.0002813.s006.docx]

| **Management domain index** | **Number of items** | **Cronbach's alpha coefficient** |
| --- | --- | --- |
| **Target setting** | 7 | 0.38 |
| **Performance monitoring** | 18 | 0.78 |
| **People management** | 20 | 0.75 |
| **Operations management** | 11 | 0.52 |
| **Financial management** | 7 | 0.95 |
| **Community engagement** | 4 | 0.64 |
