# Supplementary material for: Management practices in facilities providing HIV services to key populations in Kenya and Malawi: A descriptive analysis of management in community-based organizations
Source: PLOS Glob Public Health. 2024 Mar 20;4(3):e0002813. doi: 10.1371/journal.pgph.0002813 (PMC10954182; doi:10.1371/journal.pgph.0002813)
Supplement: S3 Table — (DOCX) [file pgph.0002813.s007.docx]

| **Variable** | **Operationalization** | **Type of variable** |
| --- | --- | --- |
| Formal education | DIC managers with a university degree. 0 = High school, some college credit, technical training. 1 = Undergraduate or Postgraduate degree" | Continuous |
| DIC size | Number of staff working at the DIC | Count |
| DIC Maturity | Number of years from DIC opening to 2019 | Count |
| Competition | Number of DICs within 30 minute-driving radio providing  HIV health services in 2019 | Count |
| DIC service scale (productivity) | Number of HIV tests provided by DIC in 2019 | Continuous |
| DIC horizontal integration | Number of HIV services provided by the DIC in 2019, where services can be prevention, treatment, or other supporting intervention. These services are: | Count |
|  | *Organization of outreach (hot spots) |  |
|  | *Outreach monthly meetings |  |
|  | *Virtual outreach (social networks/virtual spots) |  |
|  | *Prevention of mother-to-child transmission (PMTCT) |  |
|  | *General health medical check-ups (excluding testing for HIV) |  |
|  | *Screening and diagnosis for Sexually Transmitted Infections |  |
|  | *Pre-Exposure Prophylaxis (PrEP) screening and initiation |  |
|  | *Provision of condoms and lubricant |  |
|  | *Treatment for Sexually Transmitted Infections |  |
|  | *Viral load sample collection (dry blood spot (DBS) |  |
|  | *Viral load testing |  |
|  | *Viral load result collection |  |
|  | *Antiretroviral therapy (Initiation) |  |
|  | *Antiretroviral therapy (Refills) |  |
|  | *Post-Exposure Prophylaxis (PEP) screening and initiation |  |
|  | *Peer navigation to ART / retention in care |  |
|  | *Peer-to-peer positive living tracking |  |
|  | *Peer escort to DIC (e.g., during appointments) |  |
|  | *DIC monthly peer educator |  |
|  | *Mutual help groups (e.g., ART support groups, Psychosocial support groups [PSSG]) |  |
|  | *Violence prevention and response (including gender-based violence [GBV]) |  |
|  | *Legal counselling |  |
|  | *Child protection/welfare related activities |  |
| Program structure | 1 if DICs associated with IPs with two or more affiliated sites, 0 otherwise | Binary |
| Country | 1 if Malawi, 0 if Kenya | Binary |
