# Supplementary material for: Management practices in facilities providing HIV services to key populations in Kenya and Malawi: A descriptive analysis of management in community-based organizations
Source: PLOS Glob Public Health. 2024 Mar 20;4(3):e0002813. doi: 10.1371/journal.pgph.0002813 (PMC10954182; doi:10.1371/journal.pgph.0002813)
Supplement: S4 Table — Notes: P-values were calculated with the T-student test. (DOCX) [file pgph.0002813.s008.docx]

| **Management domain** | **Observations IP Unique** | **Observations IP+** | **Mean IP Unique** | **Mean IP+** | **Difference** | **p-value** |  |
| --- | --- | --- | --- | --- | --- | --- | --- |
|  |  |  |  |  |  |  |  |
| **Performance monitoring** | 13 | 32 | 83.3 | 80.6 | 2.8 | 0.624 |  |
| **People management** | 13 | 32 | 40.4 | 33.0 | 7.4 | 0.146 |  |
| **Financial management** | 13 | 32 | 78.0 | 31.3 | 46.8 | 0.001 |  |
| **Community engagement** | 13 | 32 | 69.2 | 56.3 | 13.0 | 0.168 |  |
