# Supplementary material for: Management practices in facilities providing HIV services to key populations in Kenya and Malawi: A descriptive analysis of management in community-based organizations
Source: PLOS Glob Public Health. 2024 Mar 20;4(3):e0002813. doi: 10.1371/journal.pgph.0002813 (PMC10954182; doi:10.1371/journal.pgph.0002813)
Supplement: S5 Table — Notes: Standard errors in parentheses. r2_a: Adjusted R-squared. Significantly different than zero at 99.9 (***), 99 (**), 95 (*) and 90 (+) percent confidence. # Types refers to interventions, see S3 Table. (DOCX) [file pgph.0002813.s009.docx]

| **DIC characteristics** | **Performance monitoring** | **People management** | **Financial management** | **Community engagement** |
| --- | --- | --- | --- | --- |
| **Percent of DIC managers with university degree** | 0.26 | -4.70 | -4.08 | 3.12 |
|  | (5.13) | (4.61) | (13.38) | (8.57) |
| r2_a | -.023 | .00094 | -.021 | -.02 |
| **Number of staff working at the DIC** | -0.19 | 0.25 | 1.98 | 1.28 |
|  | (0.54) | (0.49) | (1.38) | (0.88) |
| r2_a | -.02 | -.017 | .023 | .024 |
| **Number of years from DIC opening to 2019** | -1.17* | 0.94+ | 1.54 | 0.86 |
|  | (0.58) | (0.53) | (1.56) | (1.00) |
| r2_a | .066 | .047 | -.00075 | -.0062 |
| **Number of DICs within 30 minute-driving radius providing HIV health services in 2019** | 1.34 | -1.48 | -8.48+ | -3.07 |
|  | (1.97) | (1.79) | (5.02) | (3.29) |
| r2_a | -.012 | -.0074 | .04 | -.0029 |
| **Number of HIV tests provided by DIC in 2019 (thousands)** | -0.67 | -0.22 | 3.22+ | 0.82 |
|  | (0.64) | (0.59) | (1.62) | (1.08) |
| r2_a | .0019 | -.02 | .063 | -.0097 |
| **Number of HIV service types**^#^ **provided by DIC in 2019** | 1.66* | 1.34+ | 0.43 | 4.61*** |
|  | (0.76) | (0.70) | (2.09) | (1.14) |
| r2_a | .078 | .057 | -.022 | .26 |
| **Percent of DICs associated with IPs with two or more affiliated sites** | -2.78 | -7.42 | -46.77*** | -12.98 |
|  | (5.63) | (5.01) | (12.90) | (9.24) |
| r2_a | -.017 | .026 | .22 | .022 |
| **Malawi** | 1.30 | -9.67* | -28.57* | -5.00 |
|  | (5.42) | (4.71) | (13.49) | (9.05) |
| r2_a | -.022 | .068 | .073 | -.016 |
